# Comparison of the amount of affected myocardium quantified by late gadolinium enhancement and the degree of myocardial necrosis measured by cardiac troponin T in patients with myocarditis

**DOI:** 10.1186/1532-429X-15-S1-E111

**Published:** 2013-01-30

**Authors:** Florian Andre, Florian T Stock, Sebastian A Seitz, Hassan Abdel-Aty

**Affiliations:** 1 Department of Cardiology, University of Heidelberg, Heidelberg, Germany

## Background

The diagnosis of myocarditis is challenging due to its often subclinical or unspecific course. Yet, myocardial focal late gadolinium enhancement (LGE) patterns, which represent focal myocardial necrosis and imply negative clinical outcomes, can be assessed by cardiovascular magnetic resonance (CMR). Serologically, not only focal but also diffuse myocardial cell necrosis can be highly accurately detected by the cardiac-specific release of cardiac troponin T (cTnT) which has shown to have also prognostic value in these patients. However, data regarding the relationship between the focal necrosis size visualized by LGE-CMR and the serum marker of myocardial cell damage measured by cTnT are scarce. Therefore, we sought to investigate the relationship between focal LGE-CMR patterns, applying different standard deviations (std) of the LGE signal intensity, and serum levels of cTnT.

## Methods

We retrospectively included 44 myocarditis patients (39 male, 5 female). CMR images were acquired on a 1.5 T whole-body MRI (Achieva, Philips Healthcare) and LGE imaging was performed (Gadolinium-DTPA (Magnevist, Bayer Schering Pharma), 0.2 mmol/kg body weight) using an inversion-recovery SSFP sequence. Short-axis LGE images were analyzed with a dedicated scar software (cmr42, Circle Cardiovascular Imaging) which allowed the measurement of the myocardial mass affected by focal LGE applying different std of contrast enhancement (2, 3, 4, 5, 6, 8, 10 std). Values for cTnT or high-sensitive cardiac troponin T (hscTnT) where applicable were retrieved from the patients' records. Correlation analysis was performed using Pearson's r or Spearman's rho where applicable (p-value<0.05 was regarded significant).

## Results

CMR images of all patients (41.8±16.6 yrs) could be analyzed successfully and quantitatively, applying the different LGE-CMR std. Mean time difference between lab testing and CMR imaging were 0.6 days (0-4). The median values were 0.40 µg/l (0.11; 1.29) for cTnT (21 pts) and 172 pg/ml (43; 260) for hscTnT (23 pts). There was no significant correlation between serum levels of both troponin biomarkers and the LGE masses regarding all values of the applied std of contrast enhancement.

## Conclusions

The amount of LGE-CMR affected myocardium did not correlate to any degree to myocardial necrosis as measured by the cardiac-specific serum biomarker troponin T. Since inflammation of myocardial cells in myocarditis is not necessarily focally as measured by LGE-CMR, but also affects myocardium diffusely, the relative enhancement method LGE-CMR cannot entirely identify and quantify the amount of cellular inflammation. Therefore, absolute enhancement techniques like gadolinium T1-mapping could be of great interest due to their ability to measure inflammatory tissue involvement quantitatively.

## Funding

None.

